# Effects of Dietary Supplementation with Glycerol Monolaurate (GML) or the Combination of GML and Tributyrin on Growth Performance and Rumen Microbiome of Weaned Lambs

**DOI:** 10.3390/ani12101309

**Published:** 2022-05-20

**Authors:** Yi Li, Heze Wang, Yulei Zhang, Xilong Li, Xianren Jiang, Hongbiao Ding

**Affiliations:** 1 State Key Laboratory of Animal Nutrition, College of Animal Science and Technology, China Agricultural University, Beijing 100193, China; liyi__123@163.com; 2 Key Laboratory of Feed Biotechnology of the Ministry of Agriculture & Rural Affairs, Institute of Feed Research, Chinese Academy of Agricultural Sciences, Beijing 100081, China; wangheze2018@163.com (H.W.); 18061619737@163.com (Y.Z.); lixilong@caas.cn (X.L.); jiangxianren@caas.cn (X.J.)

**Keywords:** glycerol monolaurate, tributyrin, weaned lambs, growth performance, rumen microbiome

## Abstract

**Simple Summary:**

The weaning period is important for the growth and ruminal function of lambs. Therefore, novel feed additives that improve the growth performance of weaned lambs are continuously being developed and researched. On this basis, the effects of dietary supplementation with glycerol monolaurate (GML) or the combination (Solider, SOL) of GML and tributyrin (TB) on the growth performance and rumen microbiome of weaned lambs were studied. In this study, dietary supplementation with GML or SOL improved the growth performance and nutrient digestibility of weaned lambs. In addition, GML or SOL supplementation changed the rumen microbiome, such as by increasing the relative abundance of Actinobacteria and Verrucomicrobia and decreasing the relative abundance of *Ruminococcus*. In summary, the study provides new insights into the application of GML and SOL in weaned lambs.

**Abstract:**

Our objective was to evaluate the effects of dietary supplementation with glycerol monolaurate (GML) or the combination (Solider, SOL) of GML and tributyrin (TB) on the growth performance and rumen microbiome of weaned lambs. Thirty-six male Hu lambs (11.46 ± 0.88 kg BW and 40 ± 5 days of age) were divided into three treatment groups: (1) CON: basal diet, (2) GML: basal diet supplemented with GML at 1.84 g/kg DM, and (3) SOL: basal diet supplemented with SOL at 3 g/kg DM. GML increased the final BW (*p* = 0.04) and ADG (*p* = 0.02) compared with CON. There were no significant differences in the DMI (*p* > 0.10) among the three treatment groups. GML and SOL tended to decrease the dry matter intake/average daily gain (*p* = 0.07) compared with CON. GML tended to increase the apparent digestibility of CP (*p* = 0.08) compared with CON. SOL increased the apparent digestibility of NDF (*p* = 0.04) compared with CON. The Chao1 and Shannon indexes of SOL were both significantly higher than those of the other groups (*p* = 0.01). LefSE analysis showed that Bifidobacteriaceae of the Bifidobacteriales was enriched in the GML group. In addition, compared with GML, SOL reduced the relative abundance of Actinobacteria (*p* < 0.01) and increased the relative abundance of Verrucomicrobia (*p* = 0.05), and GML reduced the relative abundance of *Ruminococcus* (*p* = 0.03). Our results indicated that dietary supplementation with GML or SOL improved growth performance and feed conversion, and changed the rumen microbiome of weaned lambs.

## 1. Introduction

In intensive farming systems, early weaning can be a useful management option to reduce the overall ewe feed demand and the days of weaning to achieve liveweight targets [1]. However, weaning stress in early life may compromise health, productivity, and welfare as a result of changes in nutrients and the environment [2]. Ekiz et al. [3] reported that early weaning results in reduced lamb growth because of the physiological delay in ruminal development and slow adaptation to solid feed consumption. The weaning period is critical for rumen structure and function development in lambs. After weaning, the large intake of solid feed stimulates the rapid development of rumen papilla and musculature, and promotes the perfection of the rumen microbiome [4]. In addition, rumen bacterial colonization has a long-term impact on digestion efficiency [5]. Therefore, it is necessary to develop effective nutritional measures to improve the growth performance and rumen microbiome of weaned lambs.

Glycerol monolaurate (GML), as a naturally occurring monoglyceride, is the esterified form of lauric acid [6]. Many studies have shown that GML supplementation could have positive effects on the performance and health of broiler chickens and weaned pigs [7,8,9,10]. Fortuoso et al. [11] supplemented feed with 300 mg/kg of GML and observed increased body weight, weight gain, and daily weight gain of Cobb 500 broiler chicks, and GML showed a potent antimicrobial effect and no toxicity to the chicks. In addition, GML supplementation improved the lipid metabolism of laying hens, such as by reducing fat deposition in the abdominal adipose tissue and lowering the serum triglyceride and total cholesterol levels [12]. GML could reduce bacteria and virus infectivity in feed, which may help to curb disease transmission [8,13]. In order to evaluate the safety of GML application in calves, Wieland et al. [7] added GML at 0.006% of body mass and detected glutamate dehydrogenase (GLDH) and glucose to evaluate liver function. The results suggested that GML does not have a toxic effect on liver function in calves. On the other hand, GML exerts bacteriostatic effects by inhibiting protease activity and nutrient absorption on the membrane of Gram-positive bacteria [14]. Schlievert et al. [15] showed that GML could kill the vegetative cells and spores of aerobic *B. anthracis*, *B. subtilis*, and *B. cereus* and anaerobic *Clostridium perfringens* and *Clostridium (Clostridioides) difficile*. GML has no inhibitory effect on *Lactobacillus*, because *Lactobacillus* can synthesize Reuterin, which is the analog of GML [16]. However, we found no studies on the effect of GML on the growth performance and rumen microbiome of weaned lambs.

Tributyrin (TB), a typical butyric acid derivative, is a short-chain fatty acid ester. It can be decomposed into three molecules of butyric acid and one molecule of glycerol by the action of pancreatic lipase in the intestinal tract of animals, which can further maintain intestinal integrity, regulate the intestinal microbiome, and participate in immune regulation and energy metabolism [17,18,19]. He et al. [20] showed that TB could alleviate weaning stress in piglets by reducing the activities of alanine aminotransferase, aspartate transferase, and alkaline phosphatase. In dairy cows, TB could relieve heat stress and increase production performance by reducing the inflammatory responses of lymphocytes, and a study has shown that TB supplementation in pasteurized waste milk at 2 g/L can increase the growth performance and health of dairy calves and linearly reduce the haptoglobin, endothelin, and IL-1β concentrations [21]. Ren et al. [17] suggested that TB enhances fibrolytic enzyme activity and increases the volatile fatty acid concentration, and that TB could improve the microbial protein yield and fermentation in the rumen. 

However, the effects of dietary supplementation with GML or SOL on the growth performance and rumen microbiome of weaned lambs are unknown. The purpose of this study was to explore the effects of dietary supplementation with GML or SOL on the growth performance and rumen microbiome of weaned lambs, and to provide a scientific basis for their use as a feed additive in weaned lambs.

## 2. Materials and Methods

### 2.1. Tested Product

Monoglyceride laurate (GML, ≥93%) and Solider (SOL, monoglyceride laurate ≥57%, diglyceride laurate ≥6%, and tributyrin ≥14%) were obtained from Guangzhou Baishilu Biotechnology Co., Ltd. (Guangzhou, China).

### 2.2. Experimental Animals and Design

The study was conducted between June and August 2020 at the Sheep Industry Test Station (Yancheng, China). All lambs used in the study were of the Hu breed. A total of 36 healthy weaned lambs (BW = 11.46 ± 0.88 kg, age = 40 ± 5 d) were divided into treatment groups: control treatment (CON), GML, and SOL, with 3 replicates per treatment and 4 lambs per replicate. The experimental diets were as follows: (1) CON: basal diet, (2) GML: basal diet supplemented with GML at 1.84 g/kg DM, (3) SOL: basal diet supplemented with SOL at 3 g/kg DM. GML and SOL supplements were mixed with the basal diet. The diet was offered ad libitum. Table 1 shows the ingredients and chemical composition of the basal diet. All experimental lambs were kept in barns in their separate treatment groups and maintained in individual pens (3.0 × 1.0 m). The experiment lasted for 45 days, with a 3-day adaptation period. During the experimental period, the daily feed intake of each lamb was recorded. We weighed the initial BW and final BW of each lamb with an empty stomach at 9:00 am on 1 d and 45 d of the experimental period, respectively. We also calculated the average daily gain (ADG). The calculation formula was as follows:

Average daily gain (kg) = (final BW − initial BW) / 45.

### 2.3. Diet Sampling and Analysis

We collected diet samples twice per week and stored them at −20 °C. At the end of the experiment, they were used to analyze the composition and nutrient levels of the basal diet. We determined the DM content using oven drying at 105 °C to constant mass (method 930.15, AOAC) [22] and calculated the dry matter intake (DMI) using DM. We used Kjeldahl nitrogen analysis to determine the CP (method 945.16, AOAC) [22] and used a Soxhlet extractor to determine the EE content (method 945.16, AOAC) [22]. NDF and ADF were analyzed using heat-stable amylase (A3306, Sigma Chemical Co., St. Louis, MO, USA) and sodium sulfite according to the procedure of Van Soest [23]. In addition, we used a muffle furnace to measure the ash content by combustion (method 942.05, AOAC) [22]. We used the colorimetric method and atomic absorption spectrometry to analyze phosphorus (Spectrophotometer UV755N, Yoke Instrument Co., Ltd., Shanghai, China) and calcium (PerkinElmer AAS800, Waltham, MA, USA), respectively [24].

### 2.4. Determination of Apparent Digestibility

Feed digestibility was measured during the last 4 days of the experiment as described by Almeida et al. [25]. Two lambs with similar average body weights were selected from each replicate and moved to individual metabolism cages. A wire-screen basket was placed behind each cage for feces collection. Fresh feces were collected and weighed twice at 09:00 and 17:00 every day, and then nitrogen fixation was carried out by adding 5 mL of 10% sulfuric acid per 100 g of manure sample. After continuous collection for 4 days, all fecal samples were mixed and sampled by the quartering method. They were dried at 65 °C for 4 h and then maintained at room temperature for 24 h and placed in sample bags. The detection method for DM, OM, CP, EE, NDF, and ADF in the fecal samples was the same as that for the feed samples. Acid-insoluble ash (AIA) was used as an internal indicator to calculate the apparent digestibility of nutrients. The calculation formula is as follows:

The apparent digestibility of a nutrient (%) = 1 − bc/ad.

where a is the content of a nutrient in the diet; b is the content of a nutrient in the fecal sample; c is AIA content in the diet; and d is AIA content in the fecal sample.

### 2.5. Rumen Fluid Sampling and Analysis

Two lambs were selected randomly from each pen on the last day of the experimental period. In this way, rumen fluid was collected from 18 lambs with 6 lambs per treatment. We acquired rumen fluid samples using esophageal intubation 2 h after the morning feed. The rumen fluid was strained through four layers of cheesecloth with a mesh size of 250 μM, and was frozen in liquid nitrogen immediately until the extraction of DNA.

### 2.6. DNA Preparation and Sequencing

We extracted bacterial DNA using a PowerSoil™ DNA Isolation Kit (MO BIO Laboratories, Carlsbad, CA, USA) with TRIzol agent and used 16S rRNA primers to identify bacterial taxa (F: 5’-ACTCCTACGGGAGGCAGCA-3’; R: 5’-GGACTACHVGGGTWTCTAAT-3’), as described by Sun et al. [26]. Polymerase chain reaction (PCR) amplification was performed in a total volume of 50 μL, which contained 10 μL of buffer, 0.2 μL of Q5 High-Fidelity DNA Polymerase, 10 μL of High GC Enhancer, 1 μL of dNTP, 10 μM of each primer, and 60 ng of genomic DNA. The thermal cycling conditions were as follows: initial denaturation at 95 °C for 5 min; then 25 cycles of 95 °C for 1 min, 50 °C for 1 min, and 72 °C for 1 min; and a final extension at 72 °C for 7 min. The PCR products from the first PCR were purified through VAHTSTM DNA Clean Beads. A seTNd round PCR was then performed in a 40 μL reaction volume that contained 20 μL of 2 × PHμsion HF master mix, 8 μL of ddH2O, 10 μM of each primer, and 10 μL of the PCR product from the first step. The thermal cycling conditions were an initial denaturation at 98 °C for 30 s; followed by 10 cycles of 98 °C for 10 s, 65 °C for 30 s, min, and 72 °C for 30 s; and a final extension at 72 °C for 5 min. Finally, the PCR products were quantified using Quant-iT dsDNA HS Reagent and pooled. 

We performed high-throughput sequencing analysis of the bacterial rRNA genes using the Illumina Hiseq 2500 platform (2 × 250 paired ends) at the Personalbio Technologies Corporation (Beijing, China). In order to obtain high-quality clean tags, we performed quality filtering of the raw tags according to the QIIME (V1.7.0) quality control process [27]. The UCHIME algorithm was used to detect chimeric sequences to compare the tags with the reference database. Uparse software (Uparse v7.0.1001) was used to sequence the remaining useful tags. A representative sequence for each of the same operational taxonomic units (OTUs, sequences with >97% similarity) was screened for further annotation. We used the GreenGene Database to annotate the taxonomic information for each representative sequence. A standard sequence number was used to normalize the abundances of the OTUs. We used these normalized output data to quantify the alpha diversity and beta diversity.

### 2.7. Statistical Analyses

Data on the growth performance, apparent digestibility, and bacterial populations were analyzed by ANOVA after checking their independency, normality, and homogeneity using SPSS 21.0 (SPSS Inc., Chicago, IL, USA) as described by Gallardo et al. [28]. The Duncan multiple-comparisons test was used to compare the treatment means. We used GENESCLOUD (Personalbio Biotechnology Co., Ltd. Shanghai, China) to process and analyze the sequences, and we conducted base calling and image analysis using MiSeq control software (MCS) on a MiSeq instrument. We used the QIIME platform (v1.7) to produce the rarefaction curve and conduct OTU clustering by the UCLUST method. We calculated the Chao1, Shannon, and Simpson indexes using the Mothur software (http://www.mothur.org, accessed on 10 May 2022). We used the UniFrac metric on the QIIME platform to conduct principal coordinates analysis (PCoA) and compute the similarities (ANOSIM) of different groups. Linear discriminant analysis (LDA) Effect Size (LefSE) analysis was conducted to identify potential microbial biomarkers between groups using false discovery rate (FDR) values of 0.05 and an LDA threshold score of 3.0. This part of the workflow was calculated using the R packages MicrobiomeAnalystR [29] and vegan [30]. Differences among treatment groups were significant at *p* < 0.05, and had a tendency to be significant at 0.05 ≤ *p* < 0.10.

## 3. Results

### 3.1. Growth Performance and Apparent Digestibility

As shown in Table 2, GML supplementation significantly increased the final BW (*p* = 0.04) and ADG (*p* = 0.02) compared with the control diet. Although there was no significant difference in the DMI (*p* = 0.13) among the three treatment groups, dietary supplementation with GML and SOL tended to decrease the DMI/ADG (*p* = 0.07). As shown in Table 3, GML supplementation tended to increase the apparent digestibility of CP (*p* = 0.08) compared with CON. SOL supplementation significantly increased the apparent digestibility of NDF (*p* = 0.04) compared with CON.

### 3.2. Rumen Bacterial Diversity

**Figure 1:**
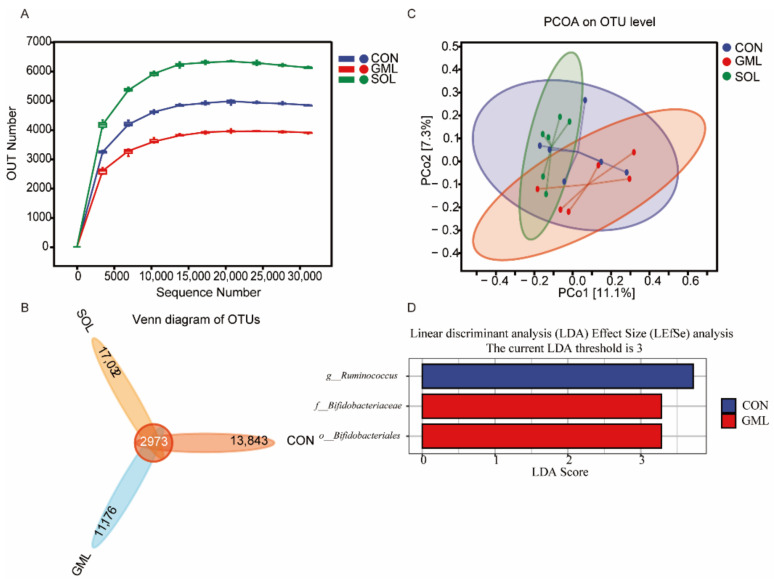
Effects of monoglyceride laurate and Solider on the rumen bacterial diversity of weaned lambs. (**A**) The rarefaction curve of sequencing based on the OTUs. OTUs, operational taxonomic units; (**B**) Venn diagram of the OTUs in the three treatment groups. OTUs, operational taxonomic units; (**C**) Principal components analysis (PCoA) of the rumen bacterial flora. The PCoA was based on the unweighted UniFrac distances between the microbiome profiles; (**D**) Linear discriminant analysis (LDA) effect size (LefSE) analyses-identified rumen bacterial biomarkers of different groups (LDA > 3, FDR < 0.05).

We analyzed a total of 1,407,846 valid sequences and 1,135,938 good sequences in the study. As shown in Figure 1A, the rarefaction curve began to flatten off. This meant that the number of OTUs did not increase when the volume of data increased, which was consistent with our sequencing data and could reflect a change in the microbiome. Additionally, as shown in Table 4, SOL showed higher Chao1 and Shannon indexes than GML (*p* = 0.01). As shown in Figure 1B, our samples generated 45,024 OTUs at 97% similarity in the three treatment groups. The number of OTUs in SOL (20,005) was higher than those in CON (16,816) and GML (14,149). Moreover, we found 13,843, 11,176, and 17,032 unique OTUs in CON, GML, and SOL, respectively. As shown in Figure 1C, PCoA axes 1 and 2 accounted for 11.1% and 7.3% of the total variation, respectively. Analysis of similarities (ANOSIM) yielded an R of 0.14 (*p* = 0.06), which indicated that a tendency for significant differences was found between each group. LefSE analysis was performed to identify the differentially abundant bacteria compositions (Figure 1D; LDA > 3, FDR < 0.05). In the CON group, *Ruminococcus* was identified as an important microbial biomarker. Bifidobacteriaceae of the Bifidobacteriales was enriched in the GML group.

### 3.3. Rumen Bacterial Composition

As shown in Table 5, Bacteroidetes and Firmicutes were the two most abundant phyla. As for the relative abundance of Bacteroidetes, compared with CON, it was increased with GML and SOL supplementation, while the relative abundance of Firmicutes decreased, but the differences among the three treatment groups were not significant. In addition, compared with GML, SOL supplementation significantly reduced (*p* = 0.03) the relative abundance of Actinobacteria. Compared with CON, SOL supplementation significantly increased (*p* = 0.05) the relative abundance of Verrucomicrobia. As shown in Table 6, compared with CON, GML supplementation significantly decreased (*p* = 0.03) the relative abundance of *Ruminococcus*.

## 4. Discussion

### 4.1. The Effect of Glycerol Monolaurate (GML) or the Combination (Solider, SOL) of GML and Tributyrin (TB) on the Growth Performance of Weaned Lambs 

In this study, dietary supplementation with GML or SOL significantly increased the daily gain, decreased the ratio DMI/ADG, and improved the growth performance of weaned lambs. There has been a study indicating that GML supplementation in the milk replacers of weaned calves can better regulate epithelial cell proliferation and probably have an “emollient effect”, leading to an easier “peeling” that might increase the efficiency of nutrient transport across the epithelium [31]. Moreover, GML is able to augment the production of globulins [32]. The effect of coconut oil, one of the main sources of GML, on performance has been extensively studied in ruminants [33,34,35,36]. Faciola and Broderick [36] showed that dietary supplementation with coconut oil increased the molar proportion of ruminal propionate, reduced the milk urea N and blood urea N concentrations, and improved the protein efficiency of dairy cows. This was consistent with the results of our present study. Another study has shown that feeding coconut oil significantly reduced daily methane by changing the metabolic activity and composition of the rumen methanogenic microbiota [33]. Therefore, we hypothesized that GML could affect performance by changing the rumen microbiome.

In this study, compared with CON, dietary supplementation with SOL significantly increased the apparent digestibility of NDF in weaned lambs, and the results suggested that TB plays a positive role in ruminal fiber degradation in weaned lambs. The proposed role of TB in the gastrointestinal tract is that TB prevents the colonization of pathogens by improving the environment of the gut for the survival and propagation of beneficial commensal bacteria [37]. This would increase organic acids, lower the pH, and produce more antimicrobial compounds. These changes would have the effects of inhibiting the proliferation of pathogenic bacteria, promotion of beneficial bacteria growth, and prevention of intestinal disease induced by pathogens or stress. A previous study has shown that pasteurized waste milk supplementation with TB increases the body weight and exerts an alleviating effect on oxidative stress and the inflammatory status of dairy calves [38]. Ren et al. [17] studied the effects of TB supplementation on the ruminal microbial protein yield, fermentation characteristics, and nutrient degradability in adult Small Tail ewes in vitro and in vivo, and their results showed that TB increased the total volatile fatty acid concentration and enhanced fibrolytic enzyme activity. Therefore, TB might exert positive effects on fiber degradation and fermentation in the rumen. This is consistent with the results of our study. In terms of the intestinal microbiome, studies have shown that TB increases the relative abundance of Firmicutes, decreases the relative abundance of Proteobacteria, *Actinobacillus*, and *Escherichia*, and exerts a beneficial effect on the development of a healthy intestinal microbiome [12,37]. However, studies on the regulating effects of TB on the rumen microbiome have not been reported.

### 4.2. The Effect of Glycerol Monolaurate (GML) or the Combination (Solider, SOL) of GML and Tributyrin (TB) on the Rumen Microbiome of Weaned Lambs 

In this study, compared with CON, SOL increased the relative abundance of Verrucomicrobia. Shen et al. [39] showed that dietary fiber supplemented with 31% non-fiber carbohydrate significantly increased the relative abundance of Verrucomicrobia and the concentration of short-chain fatty acids in the rumen of goats. Non-fiber carbohydrate supplementation enhanced the intensity of TLR 10 signaling and improved the transport of ruminal energy substances into the blood. In this study, we speculated that SOL activated the TLR 10 signaling pathway and increased the relative abundance of Verrucomicrobia, promoted nutrient absorption in the rumen, and improved the performance of lambs. 

Jiang et al. [40] showed that supplementation with *Codonopsis pilosula* increased the average daily gain and the relative abundance of Actinobacteria in the rumen of early weaned calves. There has been a study indicating that Actinobacteria produces antibiotics and inhibits the growth of major plant and soil-borne pathogens [41]. Sulak et al. [42] showed that high-concentrate diets increased the abundance of ruminal Actinobacteria. In our study, SOL supplementation caused a lower relative abundance of Actinobacteria than GML. Although feeding both GML and SOL tended to decrease the DMI/ADG, GML showed better protein digestibility, while SOL showed a better effect on detergent fiber digestibility. The underlying mechanism of action needs to be further studied. In our study, GML significantly reduced the relative abundance of ruminal *Ruminococcus* in lambs, which may be caused by the strong bactericidal effect of GML on Gram-positive bacteria [43,44]. GML exerts bacteriostatic activity mainly through destroying the microbial cell wall and biofilm, thus affecting the normal material and energy metabolism of cells [43]. *Ruminococcus* is a strictly anaerobic Gram-positive bacteria that can promote the degradation of dietary fiber in the rumen [45,46]. However, our study showed that, although GML significantly reduces the relative abundance of *Ruminococcus*, it has no negative effect on dietary fiber degradation. This might to be because GML increases the relative abundance of other fiber-degrading bacteria, such as Fibrobacteres and *Butyrivibrio*. In our study, Bifidobacteriaceae of the Bifidobacteriales was enriched in the GML group, according to the LefSE analysis. Sun et al. [47] showed that leucine supplementation increased the volatile fatty acid concentration, microbial protein, and the relative abundance of *Bifidobacterium* in the rumen of weaned lambs. Furthermore, Bifidobacteriaceae could improve the explanatory ability of rumen-resistant starch and the concentration of short-chain fatty acids (SCFAs), and improve the feed conversion of weaned lambs [48]. Therefore, the increase in the relative abundance of Bifidobacteriaceae in the GML group partly explained the decrease in the DMI/ADG in our study.

## 5. Conclusions

In this study, dietary supplementation with GML or SOL increased the growth performance of weaned lambs. In addition, GML supplementation tended to increase the apparent digestibility of CP, and SOL increased the apparent digestibility of NDF. GML or SOL supplementation also changed the rumen microbiome, such as by increasing the relative abundances of Actinobacteria, Verrucomicrobia, and Bifidobacteriaceae, and decreasing the relative abundance of *Ruminococcus*. These findings provide new insights into the application of glycerol monolaurate and compound fatty acid esters containing glycerol monolaurate and tributyrin in weaned lambs.

## Figures and Tables

**Table 1:** Composition and nutrient levels of the basal diet (as-fed basis).

Item	Content (%)
Ingredients	
Silage corn	15.00
Alfalfa hay	25.00
Corn	28.80
Soybean meal	16.50
Rapeseed meal	9.50
Wheat bran	3.00
CaHPO_4_	0.50
Limestone	0.40
NaCl	0.30
Premix ^1^	1.00
Total	100.00
Nutrient levels ^2^	
ME (MJ/kg)	11.12
CP	17.46
EE	2.32
NDF	24.56
ADF	10.45
Ca	0.66
P	0.53

^1^ The premix provided the following per kg of diet: VA 15,000 IU, VD 6000 IU, VE 500 IU, Cu 10 mg, Fe 64 mg, Se 0.4 mg, Mn 54 mg, Co 0.3 mg, and I 1.2 mg. ^2^ ME was a calculated value, while the others were measured values.

**Table 2:** Effects of monoglyceride laurate and Solider on the growth performance of nutrient substances in weaned lambs.

Item	Treatment			SEM	*p*-Value
	CON	GML	SOL		
Initial BW (kg)	11.56	11.11	11.70	0.23	0.28
Final BW (kg)	20.01 ^b^	22.26 ^a^	21.36 ^ab^	0.60	0.04
ADG (kg)	0.19 ^b^	0.24 ^a^	0.22 ^a^	7.99	0.02
DMI (kg/d)	0.68	0.72	0.71	11.05	0.13
F/G	3.59 ^x^	3.05 ^y^	3.21 ^xy^	0.11	0.07

BW = body weight; ADG = average daily gain; DMI = dry matter intake; F/G = DMI/ADG. ^a, b^ Means within a row with different superscripts differ (*p* < 0.05). ^x, y^ Means within a row with different superscripts have a tendency of difference (0.05 ≤ *p* < 0.10).

**Table 3:** Effects of monoglyceride laurate and Solider on the apparent digestibility of nutrient components in weaned lambs (%).

Item	Treatment			SEM	*p*-Value
	CON	GML	SOL		
DM	81.73	82.60	83.35	0.45	0.37
OM	82.62	84.62	83.71	0.61	0.43
CP	81.03 ^y^	89.04 ^x^	85.13 ^xy^	1.48	0.08
EE	89.15	90.01	89.23	1.21	0.96
NDF	51.43 ^b^	55.07 ^ab^	56.43 ^a^	0.87	0.04
ADF	49.85	52.15	53.33	0.98	0.36

DM = dry matter; OM = organic matter; CP = crude protein; EE = ether extract; NDF = neutral detergent fiber; ADF = acid detergent fiber. ^a, b^ Means within a row with different superscripts differ (*p* < 0.05). ^x, y^ Means within a row with different superscripts have a tendency of difference (0.05 ≤ *p* < 0.10).

**Table 4:** Effects of monoglyceride laurate and Solider on community richness and diversity indexes.

Item	Treatment			SEM	*p*-Value
	CON	GML	SOL		
Chao1	4840.52 ^b^	3895.97 ^b^	6123.24 ^a^	312.45	0.01
Shannon	9.70 ^ab^	8.96 ^b^	10.42 ^a^	0.21	0.01
Simpson	0.99	0.98	0.99	0.01	0.15

^a, b^ Means within a row with different superscripts differ (*p* < 0.05).

**Table 5:** Effects of monoglyceride laurate and Solider on the relative abundance of rumen microorganisms at the phylum level (%).

Item	Treatment			SEM	*p*-Value
	CON	GML	SOL		
Bacteroidetes	62.91	71.96	67.62	0.03	0.38
Firmicutes	33.99	24.59	28.67	0.03	0.34
Tenericutes	0.70	0.57	0.75	<0.01	0.82
Proteobacteria	0.99	0.98	0.99	<0.01	0.62
Spirochaetes	0.33	0.54	0.59	<0.01	0.76
Actinobacteria	0.26 ^ab^	0.56 ^a^	0.12 ^b^	<0.01	0.03
Synergistetes	0.33	0.09	0.37	<0.01	0.65
Fibrobacteres	0.11	0.17	0.26	<0.01	0.12
TM7	0.12	0.02	0.05	<0.01	0.17
Verrucomicrobia	0.02 ^b^	0.03 ^ab^	0.07 ^a^	<0.01	0.05

^a, b^ Means within a row with different superscripts differ (*p* < 0.05).

**Table 6:** Effects of monoglyceride laurate and Solider on the relative abundance of rumen microorganisms at the genus level (%).

Item	Treatment			SEM	*p*-Value
	CON	GML	SOL		
*Prevotella*	37.54	40.37	38.29	0.04	0.95
*Succiniclasticum*	2.99	2.96	3.66	<0.01	0.44
*Ruminococcus*	2.10 ^a^	1.11 ^b^	1.82 ^ab^	<0.01	0.03
*Oscillospira*	1.41	1.65	0.58	<0.01	0.37
*Shuttleworthia*	0.25	0.04	2.17	<0.01	0.30
*Butyrivibrio*	0.44	0.62	1.23	<0.01	0.16
*RFN20*	0.47	0.52	0.92	<0.01	0.48
*CF231*	0.88	0.34	0.44	<0.01	0.22
*Anaerovibrio*	0.70	0.49	0.35	<0.01	0.49
*Anaeroplasma*	0.50	0.43	0.53	<0.01	0.91

^a, b^ Means within a row with different superscripts differ (*p* < 0.05).

## Data Availability

The datasets used and/or analyzed during the current study are available from the corresponding author upon reasonable request. The original rumen microbial data have been uploaded to NCBI (SRP349067).
